# Crystal Structures of Pyrophosphatase from *Acinetobacter baumannii*: Snapshots of Pyrophosphate Binding and Identification of a Phosphorylated Enzyme Intermediate

**DOI:** 10.3390/ijms20184394

**Published:** 2019-09-06

**Authors:** Yunlong Si, Xing Wang, Guosong Yang, Tong Yang, Yuying Li, Gabriela Jaramillo Ayala, Xumin Li, Hao Wang, Jiyong Su

**Affiliations:** 1 Jilin Province Key Laboratory on Chemistry and Biology of Natural Drugs in Changbai Mountain, School of Life Sciences, Northeast Normal University, Changchun 130024, China; 2 Zhongke Biopharm Co., LTD, East of Beijing, Beijing 101601, China

**Keywords:** inorganic pyrophosphatase, crystal structure, catalytic mechanism, lysine phosphorylation, mass spectrometry, *Acinetobacter baumannii*

## Abstract

All living things have pyrophosphatases that hydrolyze pyrophosphate and release energy. This energetically favorable reaction drives many energetically unfavorable reactions. An accepted catalytic model of pyrophosphatase shows that a water molecule activated by two divalent cations (M1 and M2) within the catalytic center can attack pyrophosphate in an S_N_2 mechanism and thus hydrolyze the molecule. However, our co-crystal structure of *Acinetobacter baumannii* pyrophosphatase with pyrophosphate shows that a water molecule from the solvent may, in fact, be the actual catalytic water. In the co-crystal structure of the wild-type pyrophosphatase with pyrophosphate, the electron density of the catalytic centers of each monomer are different from one another. This indicates that pyrophosphates in the catalytic center are dynamic. Our mass spectroscopy results have identified a highly conserved lysine residue (Lys30) in the catalytic center that is phosphorylated, indicating that the enzyme could form a phosphoryl enzyme intermediate during hydrolysis. Mutation of Lys30 to Arg abolished the activity of the enzyme. In the structure of the apo wild type enzyme, we observed that a Na^+^ ion is coordinated by residues within a loop proximal to the catalytic center. Therefore, we mutated three key residues within the loop (K143R, P147G, and K149R) and determined Na^+^ and K^+^-induced inhibition on their activities. Compared to the wild type enzyme, P147G is most sensitive to these cations, whereas K143R was inactive and K149R showed no change in activity. These data indicate that monovalent cations could play a role in down-regulating pyrophosphatase activity in vivo. Overall, our results reveal new aspects of pyrophosphatase catalysis and could assist in the design of specific inhibitors of *Acinetobacter baumannii* growth.

## 1. Introduction

Pyrophosphate (PPi) is essential to several key metabolic steps, including the synthesis of DNA [1], carbohydrates, lipids, as well as lipid degradation [2]. Hydrolysis of PPi could release a large amount of energy (19 kJ) that is often coupled to many energetically unfavorable reactions in biological organisms [3]. However, because PPi is stable under normal conditions [4], inorganic pyrophosphatase (PPase) has evolved to specifically hydrolysis the molecule [5]. In bacteria, knocking-out PPase leads to the accumulation of PPi in the cytoplasm, and thus inhibits growth [6,7].

PPases (family I, II, and III) are broadly distributed in prokaryotes and eukaryotes [8]. Family I PPase is an abundant protein in yeast and bacteria [9,10,11]. These enzymes could be directly purified from hosts and be easily crystallized [12,13]. This greatly facilitates studies of PPase. Early work has showed that family I PPases are active over a wide range of pH values [14,15,16], and Mg^2+^, Zn^2+^, Mn^2+^ could activate PPase, whereas Ca^2+^ could inhibit the enzyme [17,18]. Specific chemical modifications of PPases indicate that some unidentified tyrosines and lysines are critical for PPase activity [19,20,21], and CD spectra show that modification of these residues does not greatly change the global fold of the enzyme [21].

Since the first crystal structure of PPase from *Thermus thermophiles* was solved [13], more than 70 PPase crystal structures have been deposited in the PDB. Family I PPases have a globular shape. Crystal structures and analytical ultra-centrifugation have demonstrated that six PPase monomers assemble into an active hexamer. The crystal structure also shows that each monomer can bind three or four divalent cations (M1, M2, M3, and M4) within the catalytic center [22,23]. This is consistent with the number of divalent cations determined using enzyme assays [24,25]. Mg^2+^ is the most common cation found there [12,15], and Mg^2+^PPi has been determined to be the actual substrate of PPases [6,26].

In the early studies of PPase, there was a debate surrounding the catalytic mechanism of PPase [26,27]. Even though Cohn. M. initially developed a model for the PPase catalytic mechanism that proposed a covalent phosphorylated enzyme intermediate [26], evidence for this intermediate was not reported until the 1970s [28]. Therefore, a new model was proposed [29] in which a water molecule was coordinated and activated by M1 and M2 as the nucleophile [30] that could attack PPi and thus destroy the covalent bond between the two Pis. In addition, fluoride, which has similar properties to hydroxide, could inhibit PPase catalytic activity, but not all of it [28]. Co-crystal structures of PPases with fluorides showed that the fluoride atoms could occupy positions of the nucleophilic water [22]. However, mass spectroscopy studies of yeast PPase, hydrolyzing PPi in H_2_^18^O implied that the enzyme formed an intermediate with Pi [31]. A MALDI-TOF MS study of PPase incubated with Pi, showed that PPase could be phosphorylated at an Asp residue. However, this phosphorylation was considered to be an artifact [32]. Overall, the potential phosphorylate site at the PPase catalytic center has yet to be identified, suggesting that it is not stable under conditions used to date.

The genus Acinetobacter is a major cause of nosocomial infections. It is increasingly associated with various epidemics and has become a widespread concern in hospitals worldwide. Acinetobacter has raised particular concerns due to the severe hospital-acquired bacterial infections they cause [33,34,35]. Over the past several years, cases of Acinetobacter infection have increased dramatically [36,37,38,39]. Therefore, understanding the structure and function of Acinetobacter PPase (AbPPase) is biomedically important.

Here, we solved four crystal structures of a family I type PPase from a strain of the pathogen *Acinetobacter baumannii*. Our structures show that a water molecule from the solvent may directly attack PPi and hydrolyze the phosphodiester bond. Mass spectroscopy studies demonstrate that a highly conserved lysine residue (Lys30) is phosphorylated. Mutation of Lys30 to Arg abolished activity. The electron density of PPase-PPi co-crystal structures shows that PPi interacts with residues and divalent cations within the catalytic center. Furthermore, monovalent cation-induced effects on AbPPase activity was investigated. Overall, our results reveal new aspects of PPase catalysis.

## 2. Results

### 2.1. Structures of AbPPase

**Figure 1:**
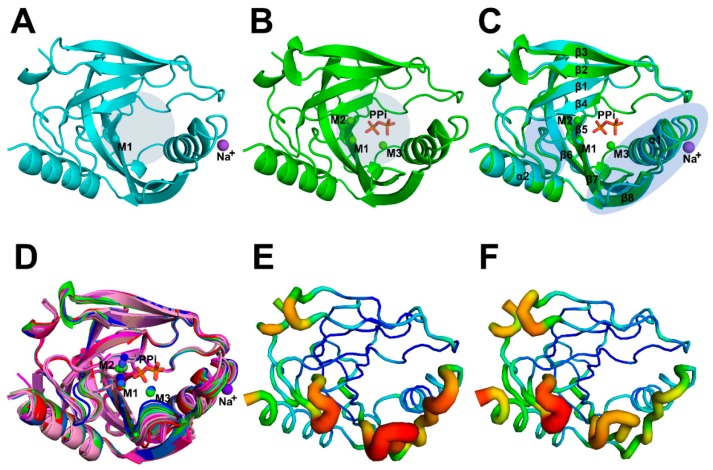
Crystal structures and B-factors analysis of WT AbPPase. (**A**) Structure 1 of AbPPase without PPi. One Mg^2+^ (M1) bound to the catalytic center, and one Na^+^ is bound to a loop close to the catalytic center. Round light gray shades represent the catalytic center position. (**B**) Structure 2 of AbPPase with three Mg^2+^ ions (M1, M2, and M3) and one PPi bound at the catalytic center. Round light gray shades represent the catalytic center position. (**C**) Alignment of the Structures 1 and 2 of AbPPase. These structures are similar to each other. However, two regions close to the catalytic center could not be perfectly merged, i.e., region 1 located between β4 and β5 and region 2 located between α1 and β8. Elliptical light purple shades represent the positions of both loops. (**D**) Alignment all of the monomers from Structures 1, 2, 3, and 4. The monomer from Structure 1 of AbPPase with Mg^2+^ is marked in red, eight monomers from Structure 2 of the AbPPase with Mg^2+^ are marked in green. The eight monomers from Structure 3 (K30R) of the AbPPase are marked in magenta. The three monomers from Structure 4 (K149R) of AbPPase with Mg^2+^ are marked in blue. Two regions close to the catalytic center also could not be perfectly merged among different monomers. (**E**) B-factor analysis of Structure 1 of AbPPase. Two loops, including region 1 located between β4 and β5 and region 2 located between α1 and β8 that around the catalytic center are flexible. (**F**) B-factor analysis of one monomer of Structure 2.

Here, we solved two crystal structures of wild-type (WT) AbPPase. The structural statistics for these structures (Structure 1 PDB: 6K21 and Structure 2 PDB: 6K27) are provided in Table 1. One was co-crystallized with PPi, and the other was not (Figure 1). The space groups of these two crystal structures are different. The crystal structures of AbPPase showed that the enzyme has a typical Rossman fold domain that is highly conserved compared to other PPases [13,22,23]. In Structure 1, we identified not only one Mg^2+^ (M1) bound to the catalytic center, but also a Na^+^ bound to a loop (residue 143–149) close to the catalytic center (Figure 1A).

Structure 2 was solved from a crystal produced in the presence of Mg^2+^ and PPi. In this structure, three Mg^2+^ ions (M1, M2, and M3) and one PPi are bound to the catalytic center (Figure 1B). Na^+^ was not detected in Structure 2. Differences in Cα RMSD values of two AbPPase structures are less than 0.8 Å, indicating that these structures are essentially the same. However, two regions close to the catalytic center could not be perfectly superposed (Figure 1C). Several highly conserved residues, including Asp66, Asp68, Asp98, and Lys143 that coordinate divalent cations or PPi, are located in these regions. Alignment of all AbPPase structures (including structures of mutants K30R and K149R) shows that these two regions could not be merged (Figure 1D). Discrepancies between these two regions in different monomers are independent of the presence or absence of PPi. In addition, the WT structure B-factors suggests that these regions are very flexible (Figure 1E,F). Overall, it appears that these flexible regions may regulate the catalytic activity of AbPPase by modifying ligand selectivity.

**Figure 2:**
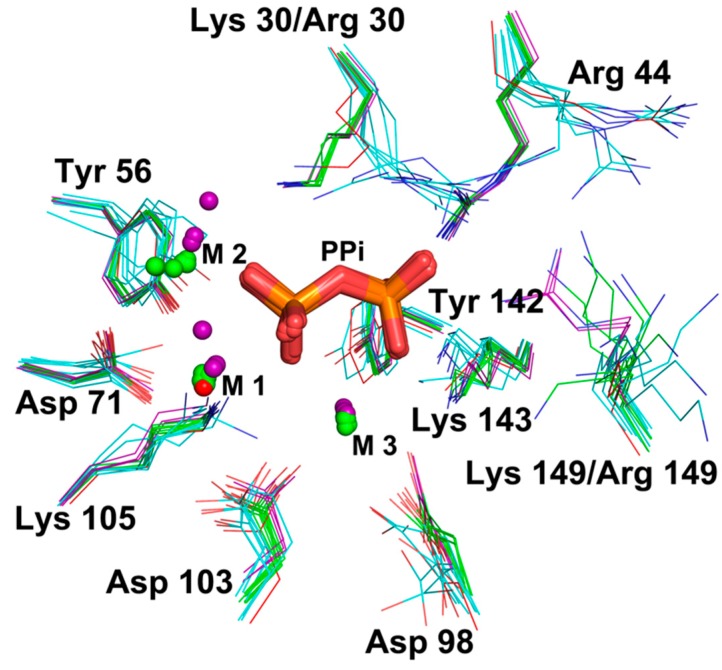
Alignment of all catalytic centers of AbPPase monomers. Structure 1 of AbPPase without PPi was marked in red, Structure 2 of AbPPase with PPi was marked with green, Structure 3 (K30R) of AbPPase without PPi was marked with cyan, and Structure 4 (K149R) of AbPPase with PPi was marked in purple.

We also solved crystal structures of mutants K30R and K149R (Structure 3 PDB: 6KI7 and Structure 4 PDB: 6KI8, respectively). The space groups of these two variants are different from those of the WT structures (Table 1). In the enzyme assay, K30R lost the ability to hydrolyze PPi. In contrast, K149R maintained the same activity as the WT enzyme (see enzyme assay section). In addition, K149R could be co-crystallized with PPi, suggesting that substitution of Lys149 to Arg did not affect the affinity of AbPPase for PPi. Differences in Cα RMSD values between monomer subunits of the two mutants compared to WT structures are less than 1 Å, indicating that both mutants have similar structures as WT AbPPase. The alignment of the catalytic centers of all monomers shows that the positions of ions M1 and M2 are different among monomers (Figure 2). However, the positions of the actual substrate PPi-M3 in different monomers are the same. The positions of Lys30, Arg44, Tyr56, Asp71, Asp98, Asp103, Lys105, Tyr142, and Lys143 in WT and K149R are also relatively the same, indicating that the conformations of these residues are crucial for binding PPi-M3. Arg30 in the K30R adopts a different conformation compared to Lys30 in the WT enzyme. The side chain of Arg30 also affects the position of Arg44 (Figure 2). Therefore, because of these differences, K30R could not be co-crystallized with PPi and also lost the ability to hydrolyze PPi (see the following section). Moreover, Lys149 in all the monomers displayed different conformations, and mutant K149R exhibited full hydrolytic activity indicates that this residue is not important for substrate binding and catalytic activity.

**Figure 3:**
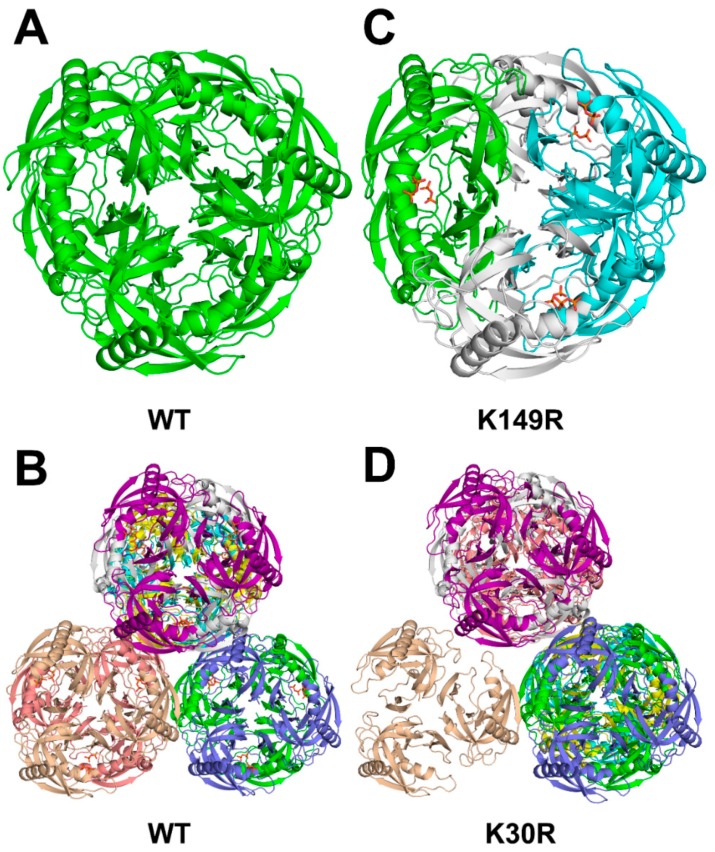
Top view of crystal packings of AbPPase. (**A**) Crystal structure of AbPPase without PPi, in which there is only one monomer in the asymmetric unit. Only one type of AbPPase hexamers was assembled. (**B**) Crystal structure of AbPPase with PPi, in which there are eight AbPPase monomers in the asymmetric unit. Eight different trimers could assemble four different types of AbPPase hexamers. (**C**) Crystal structure (K149R) of AbPPase with PPi in which there are three AbPPase monomers form a trimer in the asymmetric unit. One type of AbPPase hexamers was assembled. (**D**) Crystal structure (K30R) of AbPPase without PPi, in which there are eight AbPPase monomers in the asymmetric unit, and could assemble four different types of AbPPase hexamers and one type of trimer.

Family I PPases are hexamers formed by two stacks of trimers (Figure 3). The side view of the crystal packings of AbPPase are shown in the Supplementary Material (Figure S1). The space group of Structure 1 is P6322, and there is only one AbPPase monomer in the asymmetric unit. This indicates that all monomeric AbPPases have the same structure (Figure 3A). However, the space group of Structure 2 is H3 in which there are eight AbPPase monomers in the asymmetric unit. Structures of the three monomers in one AbPPase trimer are the same; therefore, each AbPPase monomer in Structure 2 could represent one AbPPase trimer. Eight different trimers could assemble into four different types of AbPPase hexamers with different catalytic constants (Figure 3B). The space group of Structure 4 (K149R) is P41212, with three monomers in the trimer of the asymmetric unit (Figure 3C). Thus, it could form a hexamer with the trimer from another asymmetry unit. The space group of Structure 3 (K30R) is H32 with eight monomers in the asymmetry unit (Figure 3D). Overall, the crystal packing of these four AbPPase variants is different. It appears that slight differences (residue mutations or PPi bound or not) in the catalytic center could induce a global structural change in the enzyme.

### 2.2. Variable PPi Binding Snapshots in WT AbPPase Catalytic Center

**Figure 4:**
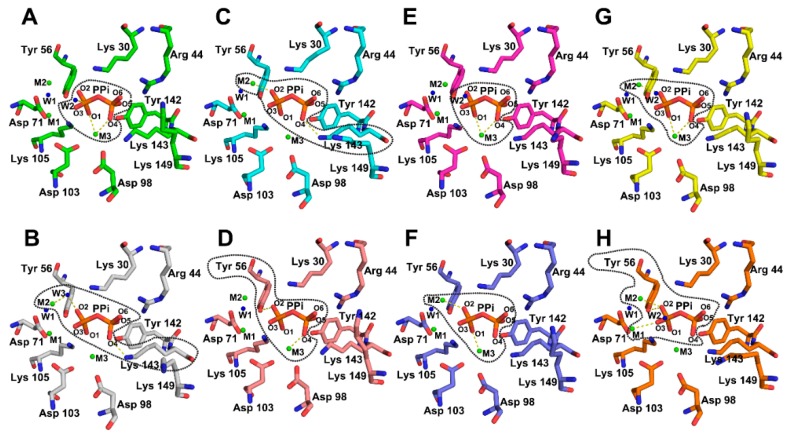
Variable PPi binding snapshots in eight AbPPase catalytic centers according to the distribution of electron densities. The dash lines delimit the regions where the electron densities of the atoms were fused with each other. Monomer (**A**,**B**), (**C**,**D**), (**E**,**F**), and (**G**,**H**) assemble into AbPPase hexamer 1, 2, 3, and 4. A water molecule (W2) from the solvent is close to the phosphate group and could be the real catalytic water (**A**). The left phosphate in PPi is named as Pi1 and the right one is named as Pi2.

The resolution of Structure 2 is 1.86 Å, which is less than inter-residue distances, divalent cations, and water molecules within the catalytic center. Therefore, the electron densities of the groups within the catalytic centers of the eight AbPPase monomers are sufficiently resolved to determine bonding networks with PPi, accurately. By carefully analyzing Structure 2, we found that the electron density of eight AbPPase catalytic centers is different (Figure 4). Each hexameric unit has two trimeric layers with different catalytic constants. AbPPase monomers in one trimer exist in the same state, indicating that two AbPPase trimers in the hexameric enzyme could be in two different states. Monomer A and B, C and D, E and F, and G and H assemble into the AbPPase hexamer 1, 2, 3, and 4 (Figure 4 and Figure S2). In monomer A, the electron density of M3 overlaps with O1 and O4 atoms of PPi, and a water molecule (W2) overlaps with O3 of PPi. The distance between W2 and Pi is only 3.3 Å, which is shorter than the distance between W1 and Pi (i.e., 4.1 Å). In monomer B, the electron density indicates that the PPi O4 atom could form a bond with the Lys143 Nζ group. In addition, the electron density of M2, a water molecule (W3) and the PPi O2 atom overlap. In monomer C, the electron densities from PPi O4 and the Lys143 Nζ group also overlap, whereas in monomer F, PPi O2 overlaps with M2. In monomer D, the electron density of M3 overlaps with PPi O4. Interestingly, PPi O2 seems to form a bond with the Tyr56 ring OH group. In monomer E, the electron density of M3 completely overlaps with PPi O1 and O4, and that of PPi O2 with M2. In monomer F, the electron density of Pi2 only partially interacts with other atoms. In contrast, Pi1 is joined to the surrounding atoms. The electron density of PPi O2 bifurcately overlaps with M2 and the Tyr 56 ring OH. On the other hand, the electron density of PPi O3 overlaps with M1. In monomer G, the electron density of PPi only overlaps with that of M3, but not with other atoms. In monomer H, the electron density of PPi O1 overlaps with M3 and PPi O2 with M2.

### 2.3. Enzyme Assay

**Figure 5:**
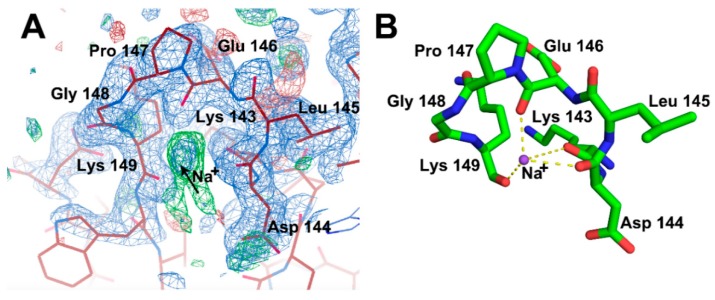
Na^+^ was identified in crystal Structure 1 of AbPPase. The 2|*F_o_*|–|*Fc*|, α_c_ map contoured at 1δ is shown as blue density. The |*F_o_*|–|*Fc*|, difference density map contoured at 3δ is shown as green density. (**A**) The electron density map of Na^+^ molecule in Structure 1 of AbPPase. Black arrow indicates where the Na^+^ molecule is located. (**B**) The coordination of Na^+^ molecule by a loop in Structure 1 of AbPPase. Lys143 and Lys149 make coordination with Na^+^ through their β-ketone groups. Pro147 forms the turn for this loop.

**Figure 6:**
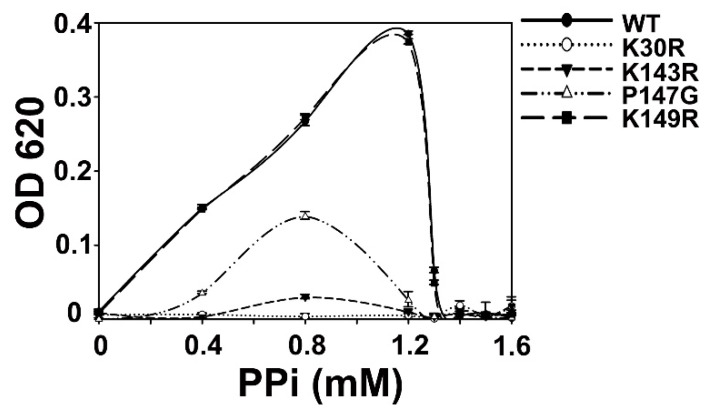
Enzyme assay of WT and four variants of AbPPase. The K149R variant had a similar activity as the WT AbPPase, K30R and K143R variants lost almost all their activity, and the P147G variant had a significantly reduced activity compared to the WT AbPPase. Error bars represent standard deviations for n = 3 independent experiments.

In Structure 1, we observed that a Na^+^ atom binds to residues within a loop of the enzyme (Figure 5). In several other PPase structures, this conserved loop could also bind Na^+^ and K^+^ [23,40]. The function of these monovalent cations in PPases was unknown. In AbPPase, β-ketone groups from two highly conserved residues, Lys143 and Lys149, coordinate with this Na^+^. Moreover, Pro147 forms the turn of that loop. In order to study the binding effects of Na^+^ on AbPPase, three variants (K143R, P147G, and K149R) were generated. Lys143 and Lys149 are also part of the catalytic center. Therefore, K143R and K149R could also be used to investigate the catalytic function of Lys143 and Lys149. Using mass spectroscopy (see the following section), we discovered that Lys30 could be phosphorylated. In order to make clear the function of Lys30, we generated the variant K30R and investigated the PPi hydrolytic activity with all the AbPPase variants (Figure 6). K149R showed a similar activity as WT AbPPase, indicating that the mutation of Lys149 to Arg had no influence on AbPPase activity. In contrast, K30R and K143R lost almost all the activity, demonstrating that Lys30 and Lys143 play key roles in AbPPase hydrolyzing activity. Furthermore, mutant P147G still maintained some activity (Figure 6).

**Figure 7:**
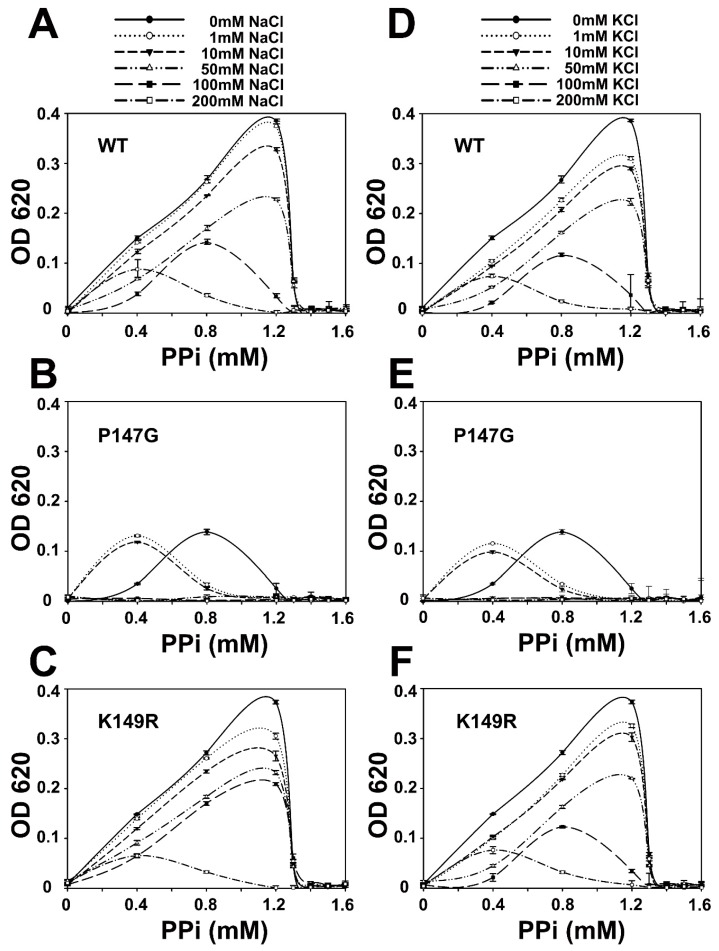
The enzyme assay inhibitory effects of Na+ and K+ on WT, P147G, and K149R of AbPPase. (**A**,**D**) Na^+^ and K^+^ could, in a concentration-dependent manner, inhibit WT of AbPPase hydrolysis of PPi, respectively. The higher the Na^+^ and K^+^ concentration, the lower the WT of AbPPase catalysis activity. (**B**,**E**) Na^+^ and K^+^ inhibit P147G of AbPPase hydrolysis of PPi, respectively. (**C**,**F**) Na^+^ and K^+^ could, in a concentration-dependent manner, inhibit K149R of AbPPase hydrolysis of PPi, respectively. K149R variant showed a similar behavior to the WT AbPPase to the inhibition of monovalent cations. Error bars represent standard deviation for *n* = 3 independent experiments.

Inhibitory effects from Na^+^ and K^+^ on WT, P147G, and K149R were also determined (Figure 7). Results show that Na^+^ and K^+^ could inhibit AbPPase activity in a concentration-dependent manner (Figure 6D and Figure 7A). As the concentration of Na^+^ and K^+^ were increased, AbPPase maximal activity appeared at a lower concentration of PPi. Interestingly, the maximum activity of P147G in the presence of these monovalent cations occurred at 0.8mM PPi. However, the maximum activity of P147G did shift to 0.4 mM when 1 mM or 10mM monovalent cations were present. Higher cation concentrations totally inhibited P147G activity, implying that P147G is more sensitive to Na^+^ and K^+^ than the WT AbPPase (Figure 7B,E). This also suggests that Na^+^ binding to AbPPase is not an artifact, but does play a regulatory role in the activity of this enzyme. K149R showed similar cation-induced behavior as the WT AbPPase (Figure 7C,F).

**Figure 8:**
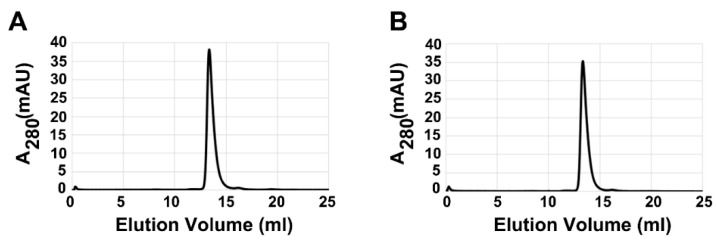
Gel filtration analysis of the global fold of AbPPase. (**A**) Gel filtration profile of AbPPase in the presence of 1.6 mM PPi and 1.6 mM MgCl_2_. The elution peak of AbPPase falls at 13.36 mL. (**B**) The gel filtration profile of AbPPase in the presence of 200 mM NaCl,1.6 mM PPi and 1.6 mM MgCl_2_. The elution peak of AbPPase falls at 13.32 mL.

It has been reported that there is an equilibrium between trimeric and hexameric forms of PPase. The trimeric form of PPase exhibited different catalytic activity compared to the WT enzyme [41,42]. Gel filtration demonstrated that Na^+^, Mg^2+^, and PPi do not greatly alter the global fold of AbPPase (Figure 8A,B). The elution profiles of AbPPase in these running buffers are the same, indicating that the inhibitory effects of Na^+^ occur via binding to the loop, rather than by altering the global structure of the enzyme.

### 2.4. AbPPase Lys30 is Phosphorylated during PPi Hydrolysis

**Figure 9:**
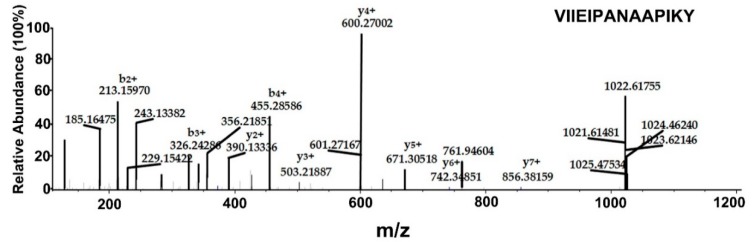
Mass spectrometry analysis of recombinant AbPPase after the in vitro PPi hydrolysis reaction to determine phosphorylation sites. A sufficient number of complementary Bn anions (N terminus-derived fragment ions) and Yn ions (C terminus-derived fragment ions) were detected to assign the phosphorylation sites to particular amino acids.

Here, we used MS to identified chemical modifications of AbPPase during PPi hydrolysis. After 10 min of the enzyme reaction, chymotrypsin was added to the reaction to digest AbPPase, and unreacted AbPPase was used as a negative control. MS identified 45 peptides, covering 98.2% of the enzyme. Our results showed that several methionine and cysteine residues could be oxidized and carbamidomethylated, respectively (Table 2). During hydrolysis, Lys30 was found to be phosphorylated (Table 2, Figure 9). In contrast, Lys30 remained unphosphorylated in the negative control experiment. This indicates that phosphorylation of Lys30 is specific to the hydrolysis reaction. However, only two phosphorylated fragments containing Lys30 were detected, suggesting that the amount of phosphorylated Lys30 is either low or the phosphorylated form of Lys30 is unstable. In the crystal structures of AbPPase, we could not identify a phosphorylated Lys30 residue. One reason for this may be the high PPi concentration needed to inhibit the enzyme activity, and Lys30 could not be phosphorylated under those conditions. Another reason might be that phosphorylated Lys30 leads to a structural change of the enzyme, thus inhibiting crystallization.

## 3. Discussion

AbPPase can reversibly catalyze the hydrolysis and synthesis of PPi, as observed with other PPases [9,10]. When the ratio of PPi to Mg^2+^ is higher than the optimized ratio, the hydrolysis activity is inhibited [43,44]. Apparently, high concentrations of PPi inhibit PPase hydrolysis activity via competitive inhibition. Biochemical and crystallographic results could validate this hypothesis. Prior to setting up our crystallization conditions, we used an enzyme assay to determine the optimal ratio of PPi to Mg^2+^ as approximately 1:1. When the ratio of PPi to Mg^2+^ was higher than 1:1, the hydrolysis activity of AbPPase was greatly diminished. With this information, we set up the crystallization conditions to contain PPi and Mg^2+^ at a ratio of 5:1. This allowed us to then co-crystal AbPPase with PPi.

There are eight AbPPase monomers in the asymmetric unit of the co-crystal structure of WT AbPPase and PPi. Interestingly, electron density maps of the catalytic centers are different (Figure S2). This indicates that PPi in the AbPPase catalytic center can fluctuate and interact with different amino acid residues, Mg^2+^ and water molecules. Therefore, it is possible that PPi could be hydrolyzed when it is trapped in a certain state. The prevalent model of PPase hydrolysis of PPi was inspired by two divalent cation-mediated catalytic mechanisms of other enzymes, such as archaebacterial flap endonucleases, ribonuclease III and PP2C Phosphatase [45,46,47]. Systematic site-directed mutagenesis studies of *E. coli* PPases have validated the importance of several amino acid residues in this model [48]. However, PPases are abundant proteins in yeast and bacteria. This enzyme could be directly purified from yeast and bacteria [9]. Early studies did not use affinity chromatography to purify overexpressed *E. coli* PPase variants. Wild-type PPase molecules may associate with various enzymes and contaminate the protein. Therefore, enzyme assay results measured by using these proteins might not be accurate.

Fluoride can mimic the nucleophilic hydroxide ions and can function as a general inhibitor of many enzymes, including phosphatases [49], enolase [50], etc. Fluoride can inhibit PPase activity [28]. Only at the saturating fluoride concentration does the initial rate of PPi hydrolysis fall to 10%, but not to zero [51]. Subsequent crystallographic studies allocated a fluoride at the position of the water molecule, which may play a role in the nucleophilic attack on the pyrophosphate group [22,52]. Aside from the model mentioned above, another model has been proposed based on very early studies of PPase. In this model, PPase could form a phosphoryl enzyme intermediate during catalysis [26]. This model was based on an ^18^O atom exchanging rate between H_2_^18^O molecules and phosphate [31]. Unfortunately, the phosphorylated enzyme intermediate was only identified after many years. In 1977, yeast PPase was digested by pepsin, and the molecular weight of peptide fragments was measured by mass spectrometry. These results showed that yeast PPase could be phosphorylated [28]. However, this phosphate was assumed to be covalently bound to Asp residues. In addition, PPase would totally lose activity after Lys and Tyr residues were chemically modified, even though modifications did not alter the overall structure of the PPase [19]. Here, we found that Lys30 could be phosphorylated during PPi hydrolysis, indicating that phosphorylation of Lys30 may be a crucial step during catalysis. After chemical modification of this residue, PPase lost activity. Moreover, phosphorylated lysine is not stable, thus possibly explaining why it has not been previously detected. In the present study, Lys30 was mutated to Arg, and this mutant lost all activity to hydrolysis PPi. The structure of K30R showed that the side chain of the Arg residue did not point in the same direction as the lysine residue in the WT enzyme (Figure 2). This suggests that Lys30 also plays a role in PPi binding.

In the prevailing model, water-activated by M1 and M2 ions could play a role in the nucleophilic attack on Pi. However, in our structure, the distance between this water molecule and Pi1 is greater than 4.0 Å, thus making this unlikely [53]. Even if the water could be activated by M1 and M2, OH^-^ may not attack pyrophosphate. In the catalytic center of K149R, M1, and M2 were not located at the same positions as M1 and M2 in the wild-type enzyme (Figure 2). But this variant still displayed almost the same catalytic activity as WT AbPPase. In addition, other reports have shown that F^-^ cannot totally inhibit PPase activity [51], suggesting that OH^-^ from the solvent may play a role as a nucleophile. In our structure, we found water (W2) 3.3 Å distant from pyrophosphate O3. This water could directly attack pyrophosphate Pi1. W2 was not coordinated to any amino acid residue or divalent cation, suggesting that this water molecule directly comes from the solvent. Cohn et al.’s model was proposed based on investigations of the phosphate-water exchange reaction catalyzed by inorganic pyrophosphatase from yeast [26]. In that model, ^18^O from H_2_^18^O could replace ^16^O atoms in phosphate. P^16^O_4_, P^18^O^16^O_3_, P^18^O_2_^16^O_2_, P^18^_3_O^16^O, P^18^O_4_ were identified in the solution. In addition, the catalytic center of PPase is full of divalent cations and basic amino acid residues. The function of this catalytic center may only be to stabilize the catalytic intermediate and accelerate hydrolysis.

Site-directed mutagenesis has already shown that mutation of key residues could cause the hexameric form of PPase to disassociate to the trimeric form, thus altering hydrolytic activity [41]. The recovery experiment showed that when this trimeric form of PPase reverted to its hexameric form, the enzyme regained catalytic activity [42]. This indicates that the hexameric form is crucial to catalysis. Here, we identified another mechanism that could regulate catalysis. In Structure 1, we identified a Na^+^ bound to a loop, as observed in other PPase structures [23,40]. Since this loop is close to the catalytic center, this Na^+^ might regulate hydrolysis activity by modifying the conformation of the loop. We found that the presence of Na^+^ could attenuate the hydrolytic activity of AbPPase. In Structure 2 with substrate PPi, Na^+^ was not observed, indicating that substrate and Na^+^ binding may interactively regulate enzyme activity. β-ketone groups of Lys143 and Lys149 were found to coordinate with a Na^+^ ion. While mutation of Lys143 to Arg totally abolished catalytic activity, mutation of Lys149 to Arg did not influence activity. K149R was also sensitive to monovalent cation-induced inhibition like WT AbPPase. This implies that these mutations do not influence the coordination of Na^+^ to AbPPase. Proline and glycine are often located at turns in protein structures. Because Pro147 forms a turn within the active site loop of the enzyme, we mutated Pro147 to Gly and discovered that P147G is more sensitive to monovalent cations than WT AbPPase. This implies that monovalent cations could regulate the catalytic activity of AbPPase in vivo.

In summary, we solved four crystal structures of AbPPase. Alignment of the four structures indicates that PPi binding at the catalytic center of AbPPase can induce a global structural change. Structural and biochemical analysis of apo AbPPase shows that the binding of a Na^+^ ion to residues within a loop close to the catalytic center could inhibit the enzyme reaction. We also found that a highly conserved lysine residue (K30) in the AbPPase catalytic center is phosphorylated during PPi hydrolysis, and mutation of this lysine abolishes catalytic activity, suggesting that phosphorylated K30 is a catalytic intermediate. This is the first evidence that there is a phosphorylated intermediate in PPi hydrolysis. Moreover, the electron density of the catalytic center of AbPPase revealed that the PPi molecule is not static. Overall, our results reveal new mechanistic characteristics of pyrophosphatase from *Acinetobacter baumannii*.

## 4. Materials and Methods

### 4.1. Cloning, Protein Expression, and Purification

The AbPPase gene was amplified from an *Acinetobacter baumannii* strain by using primers that contain NdeI and XhoI restriction sites. PCR products were digested and cloned into a pET28a vector (Novagen, Gibbstown, USA). For overexpression of recombinant proteins, the construct was transformed into *E. coli* BL21(DE3) cells and grown in LB medium supplemented with kanamycin (100 μg/mL). When the optical density of the cultures reached 0.6–0.8, IPTG was added to a final concentration of 0.5 mM to induce protein expression. After 16 h of induction at 25 °C, the cells were harvested by centrifugation and lysed by sonification in a lysis buffer consisting of 10 mM Tris/HCl, pH 7.5, 300 mM NaCl, 2 mM β-mercaptoethanol, 20 mM imidazole. The clarified cell extract was used for protein purification with Ni-NTA Agarose (Qiagen, Hilden, Germany). After purification, the His-tagged protein was dialyzed in 10 mM Tris-HCl, pH 7.5, dissolved in deionized water. During dialysis, thrombin was added to remove the His-tag with five units (National Institutes of Health unit) per milligram protein. As determined by sodium dodecyl sulfate-polyacrylamide gel electrophoresis (SDS-PAGE), protein purity was >90%. Finally, the protein was concentrated to 20 mg/mL and stored at −80 °C.

### 4.2. Site-Directed Mutagenesis

Site-directed mutagenesis of AbPPase was performed according to the manual in the QuickChange XL site-directed mutagenesis kit (Stratagene, La Jolla, Canada). The primers for site-directed mutagenesis are as follows, K30R variant forward: 5′- GCAAACGCAGCGCCGATTAGATACGAAATCGACAAAGAT-3′, K30R variant reverse: 5′- ATCTTTGTCGATTTCGTATCTAATCGGCGCTGCGTTTGC-3′, K143R variant forward: 5′- CACTTCTTCAGCCACTACAGAGACCTGGAACCGGGCAAA-3′, K143R variant reverse: 5′- TTTGCCCGGTTCCAGGTCTCTGTAGTGGCTGAAGAAGTG-3′, P147G variant forward: 5′- CACTACAAAGACCTGGAAGGTGGCAAATGGGTCAAAATT-3′, P147G variant reverse: 5′- AATTTTGACCCATTTGCCACCTTCCAGGTCTTTGTAGTG-3′, K149R variant forward: 5′- AAAGACCTGGAACCGGGCAGATGGGTCAAAATTAGCGGT-3′, K149R variant reverse: 5′- ACCGCTAATTTTGACCCATCTGCCCGGTTCCAGGTCTTT-3′. All constructs were checked by DNA sequencing. For overproduction of the AbPPase variants, constructs were transformed into *E. coli* BL21 (DE3) cells and grown in LB medium. Purifications of the AbPPase variants were performed in the same way as for the wild-type protein. As determined by SDS-PAGE, all protein purities were >90%. Proteins were concentrated to approximately 20 mg/mL and stored at −80 °C.

### 4.3. Enzyme Assay

The enzyme activity of AbPPase and its mutants were determined spectrophotometrically by monitoring the release of free phosphate (Pi) from sodium pyrophosphate as substrate, similarly as previously described [54]. The basis of the colorimetric detection is the orthophosphate will react with ammonium molybdate to form phosphomolybdic acid. The phosphomolybdic acid is then reduced by FeSO_4_ in a weak acid solution. The blue color produced could be measured at 620 nm. The AbPPase reaction buffer contained 50 mm Tris-HCl (pH 7.5), 1.6 mM MgCl_2_, and 1.6 mM Na_4_PPi. The reaction was started by the addition of enzyme, incubate at 25 °C for 10 min. After the reaction, the color mixtures including 1% (M/V) ammonium molybdate, 5% (M/V) ferrous sulfate, and 1 N H_2_SO_4_, were added into the enzyme reaction solution. The mixture was incubated at 25 °C for 10 min. After that, the solution was monitored in a spectrophotometer, and the absorption at A620 nm was recorded. The inhibitor effects of Na^+^ and K^+^ on AbPPase were also determined. The data presented here were from three repeated experiments.

### 4.4. Gel Filtration Chromatography

Gel filtration was performed at 25 °C using the Äkta purifier 10 system (GE Healthcare, Uppsala, Sweden), a control group with a running buffer containing Mg^2+^ and PPi but without Na^+^, and a test group with a running buffer containing Na^+^, Mg^2+^, and PPi. Before protein samples were loaded onto the Superdex 200, 10/300 column, they were incubated at 25 °C for 10 min, and then eluted at a flow rate of 0.3 mL/min. The absorbance was monitored at 280 nm.

### 4.5. Crystallization, Data Collection, and Structure Determination

Hampton Research packs (PEGRx1, PEGRx2, Index, Crystal Screen, and Crystal Screen 2) were used for the initial crystallization screen (sitting-drop vapor diffusion method). To obtain a crystal suitable for X-ray diffraction, we optimized crystallization by using the initial three conditions. Larger crystals were obtained from drops that contained 1 μL protein and 1 μL solution from the well containing 2.2 M DL-malic acid, pH 6.5, with or without 5 mM PPi (hanging-drop method). The crystallization conditions of the mutants are similar to the wild-type. Prior to X-ray data collection, crystals were soaked for approximately 1 min in the reservoir solution supplemented with 20% (*v*/*v*) glycerol as a cryoprotectant, and then flash cooled in liquid nitrogen.

Data sets were collected at 100 K at the Shanghai Synchrotron Radiation Facility (Shanghai, China). Data sets were indexed and integrated using XDS [55] and scaled using Aimless from the CCP4 package [56]. Structures were determined by Phaser [57] with a molecular replacement method using the structure of PPase (PDB: 1i6t) as the search model. Structure refinement and water updating were performed using Phenix refine [58] and Coot [59] manual adjustment. The final structure validations were performed using MolProbity [60]. Figures for all structures were generated using PyMOL [61] or Coot.

### 4.6. Mass Spectroscopy

Sample preparation for MS spectrometry was performed as previously reported with minor modifications [62]. Briefly, following the PPi hydrolysis reaction with 100 μg AbPPase, samples were dissolved in 50 mM NH_4_HCO_3_ buffer, and solely the AbPPase sample was used as a negative control in this mass spectroscopy study. Then, samples were reduced with 10 mM DTT at 37 °C for 2.5 h, followed by alkylation with 60 mM iodoacetamide at room temperature in the dark for 40 min. Afterward, the samples were loaded to an ultrafiltration column with the molecular weight cutoff of 10 kDa and centrifuged. The membrane was washed three times in UA buffer (8 M urea, 100 mM Tris-Cl, pH 8.5), then changed to 50 mM NH_4_HCO_3_ and washed twice. Finally, proteins were resuspended in 50 mM NH_4_HCO_3_ and 2 μg chymotrypsin dissolved in 50 mM acetic acid was added at an enzyme/substrate ratio of 1:50 (*w*/*w*) and incubated at 37 °C overnight. The digestion was stopped by adding TFA at a final concentration of 0.4% (*v*/*v*). The sample was desalted using a homemade C18 solid-phase extraction column, the column was washed three times, with 200 µL 0.2% TFA buffer and the eluate with 80% acetonitrile two times. Elutes were combined and dried in a speedvac for subsequent LC-MS/MS analysis.

LC-MS/MS was performed on an EASY-nLC 1200 system coupled to a Q-Exactive mass spectrometer equipped with a nano-electrospray ion source (Thermo Scientific, USA). In brief, chymotrypsin peptide mixture were loaded onto an Acclaim PepMapTM 100 column (C18, 100 μm × 2 cm, 5 μm, 100 Å) and then separated on an Acclaim PepMapTM RSLC column (C18, 50 µm × 15 cm, 2 μm, 100 Å) with a linear gradient of 5–30% B in 80 min, to 40% B in 16 min and then to 100% B in 8 min at a flow of 300 nL/min. The mobile phase A was 0.1% formic acid in water, and mobile phase B was 0.1% formic acid in acetonitrile: water (80: 20, *v*/*v*). The mass spectrometer was operated in a data-dependent mode, which could automatically switch between MS and MS/MS acquisition. Full MS scans were acquired in the range of *m*/*z* 350–2000 at a resolution of 70,000. The AGC target value was set at 3e6 with maximum injection time of 50 ms. The 15 most abundant precursor ions were considered for fragmentation using higher-energy collisional induced dissociation with a normalized collision energy of 27. The AGC target value of MS/MS scan was set at 1e5, and the resolution was 17,500. The data acquisition was performed by Xcalibur software (version 2.1, Thermo Scientific, MA, USA).

All LC-MS/MS data were searched using the Proteome Discoverer software (version 2.2, Thermo Scientific, MA, USA). The search parameters were set as follows: Only fully chymotrypsin peptides with no more than two missed cleavages were considered. Precursor ion mass tolerance was 10 ppm, and fragment ion mass tolerance was 0.02 Da. Phosphorylation was selected as dynamic modifications at the peptide of K and Y while cysteine carbamidomethylation was set as a fixed modification. Spectra were searched against the peptide sequence of AbPPase.

## Figures and Tables

**Table 1:** Data collection and refinement statistics.

	Structure 1	Structure 2	Structure 3	Structure 4
	WT	WT	K30R	K149R
PDB Code	6K21	6K27	6KI7	6KI8
Resolution (Å)	19.90–2.00 (2.05–2.00)	19.76–1.86 (1.89–1.86)	19.96–2.75 (2.88-2.75)	19.99–1.79 (1.83–1.79)
Space group	P6322	H3	H32	P41212
Unit cell parameters (a, b, c) (Å), (α, β, γ) (°)	(102.81, 102.81, 100.78), (90.0, 90.0, 120.0)	(110.29, 110.29, 302.49), (90.0, 90.0, 120.0)	(113.01, 113.07, 551.80), (89.71, 90.00, 119.98)	(116.75, 116.75, 109.70), (90.00, 90.00, 90.00)
No. of measured reflections	212121 (15591)	572102 (29913)	350097 (44349)	837007 (49037)
No. of unique reflections	21726 (1576)	114729 (5740)	35879 (4703)	71750 (4208)
Completeness (%)	99.8 (100.0)	99.5 (100.0)	99.6 (100.0)	99.9 (100.0)
Multiplicity	9.8 (9.9)	5.0 (5.2)	9.8 (9.4)	11.7 (11.7)
Rmerge (%)	17.0 (42.0)	9.0 (95.3)	12.00 (95.4)	4.9 (56.5)
<I/δ(I)>	9.0 (4.7)	8.4 (1.7)	7.6 (2.1)	29.2 (4.5)
Rmodel (%)	18.0	23.20	29.15	15.85
Rfree (%)	21.6	25.80	32.44	18.23
Rmsd bond lengths (Å)	0.009	0.01	0.004	0.007
Rmsd bond angles (°)	0.908	0.85	0.71	0.852
Ramachandran plotf residues in favored regions (%)	98.25	98.39	92.76	98.84
Substrate/Ligand	Mg^2+^/Na^+^	PPi/Mg^2+^	-	PPi/Mg^2+^

**Table 2:** Peptides detected by mass spectroscopy.

Sequence	Modification
DVLVVTPY	
SEDGDPLDVL	
SEDGDPLDVLVVTPY	
SHYKDLEPGKW	
SPLYKDVKEY	
TDLPQLL	
VDRFMGTAMF	
VDRFMGTAMF	1 × Oxidation [M]
VDRFMGTAMF	2 × Oxidation [M5; M9]
PVAAGSVIRCRPVGKL	1 × Carbamidomethyl [C10]
VDRFMGTAMFY	
VKISGWEGADVAKAEVIKAIEAAK	
VIIEIPANAAPIKY	1 × Phospho [K13]
VIIEIPANAAPIKYEIDKDSDALF	
VPNTLSEDGDPL	
VPNTLSEDGDPLDVL	
VVTPYPVAAGSVIRCRPVGKL	1 × Carbamidomethyl [C15]
VVTPYPVAAGSVIRCRPVGKLNMEDDGGIDAKL	1 × Carbamidomethyl [C15]
VDRFMGTAMFY	1×Oxidation [M9]
NNIPAGKDAPNDIYVIIEIPANAAPIKY	
NNIPAGKDAPNDIY	
NMEDDGGIDAKLIAVPHEKLSPL	1 × Oxidation [M2]
EGADVAKAEVIKAIEAAK	
EIDKDSDALF	
EIDKDSDALFVDRF	
FVDRFMGTAMF	
FVDRFMGTAMF	1 × Oxidation [M]
GYVPNTLSEDGDPL	
IAVPHEKL	
IAVPHEKLSPL	
IAVPHEKLSPLY	
LINQVEHF	
LINQVEHFF	
INQVEHFFSHY	
MGTAMFYPANY	
MGTAMFYPANY	1 × Oxidation [M]
NMEDDGGIDAKL	
NMEDDGGIDAKL	1 × Oxidation [M2]
NMEDDGGIDAKLIAVPHEKL	
VVTPYPVAAGSVIRCRPVGKLNMEDDGGIDAKL	1 × Carbamidomethyl [C15]; 1 × Oxidation [M23]
YKDVKEY	

## Supplementary Materials

The following are available online at https://www.mdpi.com/1422-0067/20/18/4394/s1.

## Abbreviations

PPi	Pyrophophate
PPase	pyrophosphatase
AbPPase	*Acinetobacter baumannii* pyrophosphatase
W	Water molecule
M	Metal magnesium ion
WT AbPPase	Wild-type of *Acinetobacter baumannii* pyrophosphatase
MS	Mass spectrometry
