# Supplementary material for: Crystal Structures of Pyrophosphatase from Acinetobacter baumannii: Snapshots of Pyrophosphate Binding and Identification of a Phosphorylated Enzyme Intermediate
Source: Int J Mol Sci. 2019 Sep 6;20(18):4394. doi: 10.3390/ijms20184394 (PMC6770254; doi:10.3390/ijms20184394)
Supplement: Supplementary file 1 [file ijms-20-04394-s001.pdf]

International Journal of *Molecular Sciences*

MDPI

Article

Crystal Structures of Pyrophosphatase from *Acinetobacter baumannii*: Snapshots of Pyrophosphate Binding and Identification of a Phosphorylated Enzyme Intermediate

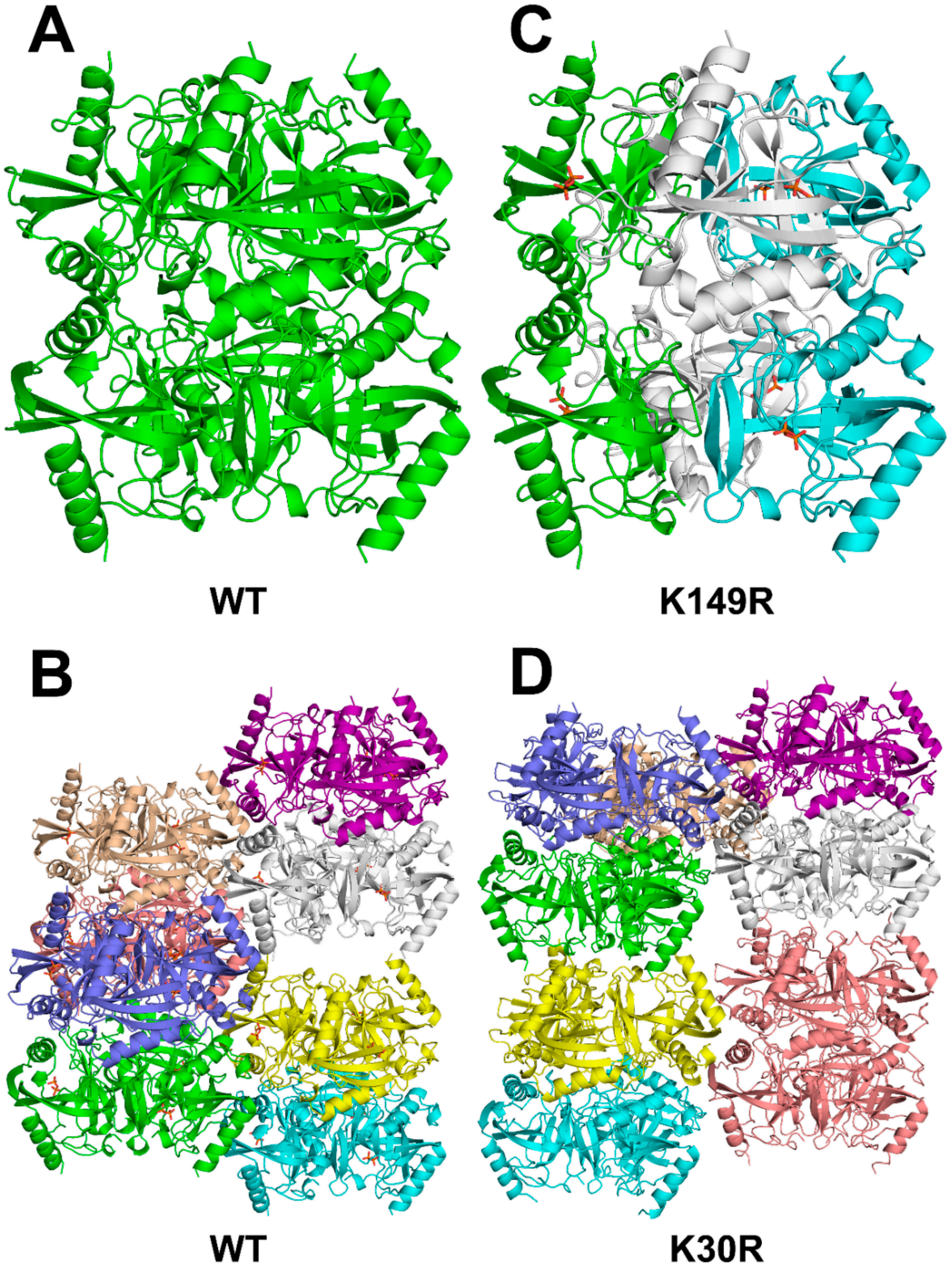

Figure S1. Side view of crystal packings of AbPPase. (**A**) Crystal structure 1 of AbPPase without PPi in which there is only one monomer in the asymmetric unit. And only one type of AbPPase hexamers was assembled (**B**) Crystal structure 2 of AbPPase with PPi in which there are eight

AbPPase monomers in the asymmetric unit. Eight different trimers could assemble four different types of AbPPase hexamers. (**C**) Crystal structure 3 (K149R) of AbPPase with PPi in which there are three AbPPase monomers form a trimer in the asymmetric unit. And one type of AbPPase hexamers was assembled. (**D**) Crystal structure 4 (K30R) of AbPPase without PPi in which there are eight AbPPase monomers in the asymmetric unit. And could assemble four different types of AbPPase hexamers and one type of trimers.

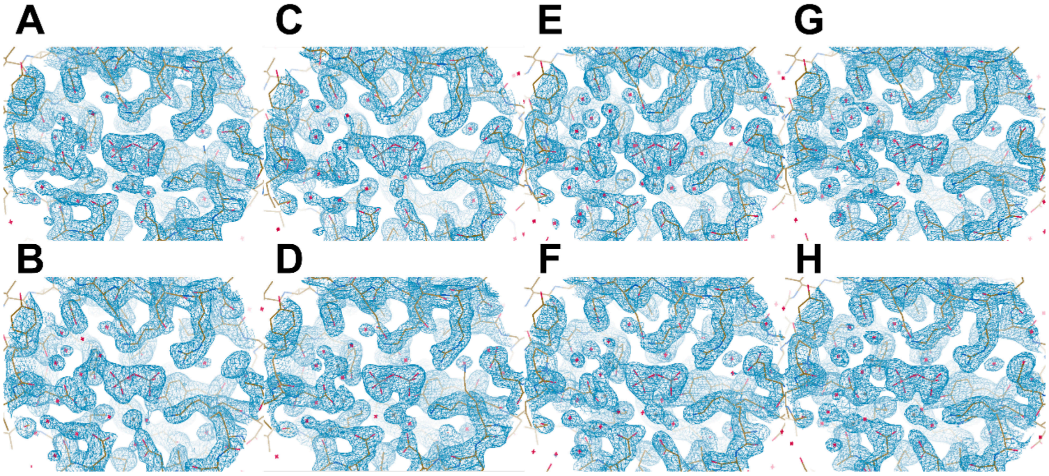

Figure S2. Comparison of the electron density for the ligand PPi molecule in each monomer subunit. (**A**) PPi molecule in monomer A subunit. (**B**) PPi molecule in monomer B subunit. (**C**) PPi molecule in monomer C subunit. (**D**) PPi molecule in monomer D subunit. (**E**) PPi molecule in monomer E subunit. (**F**) PPi molecule in monomer F subunit. (**G**) PPi molecule in monomer G subunit. (**H**) PPi molecule in monomer H subunit. The electron density for the PPi molecules showed them to be different in each monomer subunit.
